# Composite functional metasurfaces for multispectral achromatic optics

**DOI:** 10.1038/ncomms14992

**Published:** 2017-04-05

**Authors:** Ori Avayu, Euclides Almeida, Yehiam Prior, Tal Ellenbogen

**Affiliations:** 1 Department of Physical Electronics, Fleischman Faculty of Engineering, Tel-Aviv University, 69978 Tel-Aviv, Israel; 2 Department of Chemical Physics, Weizmann Institute of Science, 76100 Rehovot, Israel; 3 Tel-Aviv University Center for Light-Matter-Interaction, Tel Aviv 76100, Israel

## Abstract

Nanostructured metasurfaces offer unique capabilities for subwavelength control of optical waves. Based on this potential, a large number of metasurfaces have been proposed recently as alternatives to standard optical elements. In most cases, however, these elements suffer from large chromatic aberrations, thus limiting their usefulness for multiwavelength or broadband applications. Here, in order to alleviate the chromatic aberrations of individual diffractive elements, we introduce dense vertical stacking of independent metasurfaces, where each layer is made from a different material, and is optimally designed for a different spectral band. Using this approach, we demonstrate a triply red, green and blue achromatic metalens in the visible range. We further demonstrate functional beam shaping by a self-aligned integrated element for stimulated emission depletion microscopy and a lens that provides anomalous dispersive focusing. These demonstrations lead the way to the realization of ultra-thin superachromatic optical elements showing multiple functionalities—all in a single nanostructured ultra-thin element.

## Abstract (web summary)

Metasurfaces promise the possibility of thin optical elements, like lenses, but these mostly suffer from chromatic aberrations. Here, the authors demonstrate a multilayer fabrication approach with multiple materials to realise achromatic lenses and multispectral functional optical elements.

Some of the most important technological developments to date rely on the ability of optical elements to control and manipulate the flow of light. While for many years, mostly the functionalities of the optical elements, for example, focusing or beam shaping characteristics, were emphasized, modern technological developments pose strict requirements on the size and thickness of optical elements that are to be used in device applications. Nanotechnology in general and metamaterials or metasurfaces, in particular, provide a set of tools that paves the way towards these goals^1^,^2^,^3^.

The introduction and development of metasurfaces^2^,^3^ have been among the most important developments in the design of optical components in recent years. These are ultra-thin films, usually a few tens of nanometres thick, composed of dense subwavelength arrays of metallic^4^,^5^ or high-index dielectric^2^,^3^,^6^ resonant scatterers (nanoparticles or nanocavities) that are specifically designed for phase and amplitude control of light. Thus various metasurface lenses^6^,^7^,^8^,^9^,^10^,^11^,^12^, beam shapers^13^,^14^,^15^,^16^, optical switches^17^,^18^ and even holograms^10^,^19^,^20^,^21^,^22^,^23^,^24^,^25^,^26^,^27^ were demonstrated. Furthermore, nonlinear beam generation and beam shaping were studied^28^,^29^,^30^,^31^,^32^,^33^,^34^,^35^,^36^, including nonlinear focusing^31^,^32^,^35^ and holography^34^,^36^,^37^

A lingering problem of metasurface-based optical elements is their strong chromatic aberrations that originate from their diffractive nature. Hence, most of the designs are suitable for only a single wavelength at a specific polarization. Thus, the demonstration of broadband, polarization-independent functional elements remains a major challenge. Recently, phase-gradient achromatic metasurfaces have been demonstrated, including dual-wavelength chromatic aberration correction in the near infrared^38^,^39^,^40^. The efficiency of these metasurface lenses depends, of course, on the wavelength, and as an example, the polarization insensitive design presented by Arbabi *et al*.^40^ demonstrated efficiencies of 65% and 22% at 1,550 nm and 915 nm, respectively. The working principle behind those metasurfaces is that each nanoresonator or unit cell compensates for the phase shifts introduced during light propagation for all desired wavelengths. However, the design of such metasurfaces for arbitrary frequency bands in the visible regime has proven to be quite challenging.

Here we propose a conceptually simple yet powerful nanotechnology-driven approach for functional spectral multiplexing of broadband visible light. In our stacked multilayered metasurfaces, each layer is fabricated of different materials and with different design parameters to optimize it for a specific frequency band, and if so desired, for a predefined functionality. The layers consist of metallic disc-shaped nanoparticles that support localized surface plasmon resonances in the visible part of the spectrum. The dependence of the localized surface plasmon resonances on the parameters of the nanodiscs and on their material provides control over the spectral response of the layer so that each one operates independently and with minimal spectral crosstalk with the others. We show that multilayer elements can therefore be designed using simple design rules, and fabricated with readily available nano-lithography processes, facilitating the realization of high-performance, multifunctional elements. With this approach, broadband and multifunctional operation can be achieved simply by adding layers. This is a major advantage as opposed to current single-layer spatial multiplexing schemes, where the functionality capacity density is limited and requires fabrication of complex metasurface building blocks, especially in the visible, which limits their optical behaviour. We use this approach to demonstrate an aberration-corrected metamaterial-based triplet lens for red, green and blue (RGB) colours in the visible spectrum, integrated elements for stimulated emission depletion (STED) microscopy, and elements with anomalous dispersive focusing.

## Results

### Achromatic multilayer lens

**Figure 1:**
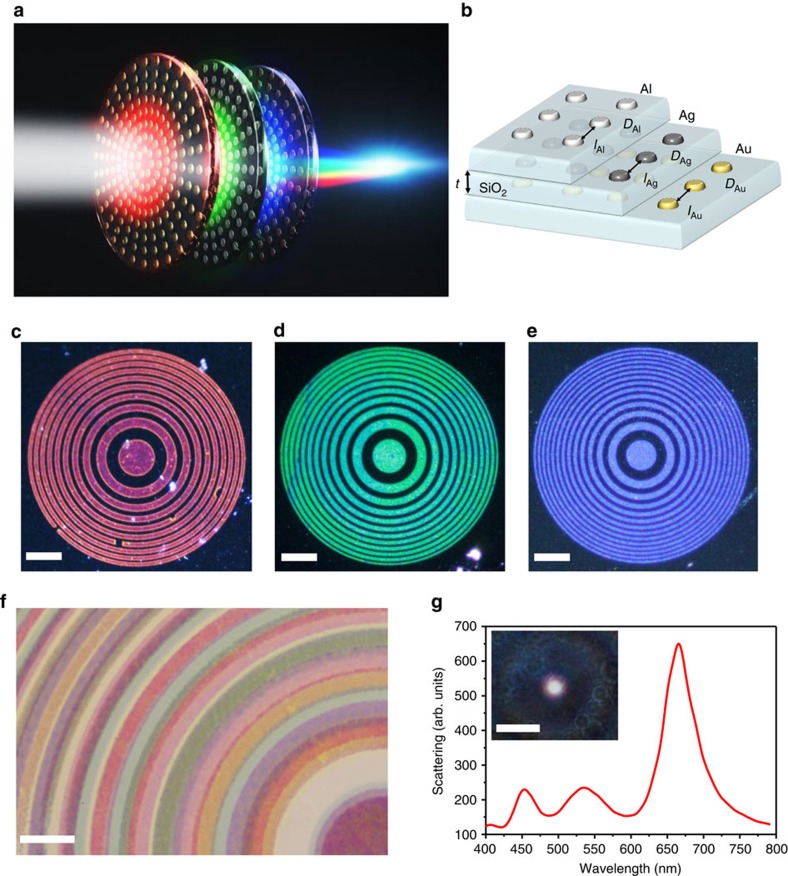
Three-layer lens. (**a**) Artist's view of the three-layer lens. When illuminated with white light, each layer focuses its designated part of the spectrum to a distance of 1 mm along the optical axis. (**b**) Schematic illustration of the layered structure (scanning electron microscope images of different layers are given in the Supplementary Fig. 2). Each layer consists of nanodiscs with the following diameters *D* and separations *l*: *D*_Au_=125 nm, *l*_Au_=185 nm; *D*_Ag_=85 nm, *l*_Ag_=195 nm; *D*_Al_=120 nm, *l*_Al_=150 nm. (**c**–**e**) Dark-field images of the single-layer lens elements. The different elements are designed to focus red, green or blue to 1 mm focal distance along the optical axis (scale bar, 35 μm). (**f**) Bright-field transmission image of the three-layer lens. The rings in each layer block a different colour, and the darker areas correspond to regions where the zones overlap (scale bar, 10 μm). (**g**) Spectrum taken under white light illumination at the focal spot, revealing the RGB components. The inset shows a coloured photograph of the obtained white focal spot at 1 mm along the optical axis (scale bar, 20 μm).

The concept of an aberration-corrected multilayer composite structure is illustrated in Fig. 1a. The lens consists of three closely stacked metasurfaces, each composed of nanoantennas made of different metal: gold, silver and aluminium, and is designed to optimally interact with light at wavelengths of 650 nm, 550 nm and 450 nm, respectively. Each of the layers acts as a narrow band binary Fresnel zone plate (FZP) lens that focuses its targeted light to the common focal point. Within each layer, the nanoparticles are closely spaced to avoid diffraction-grating effects. For the presented lenses, an interlayer distance of 200 nm was chosen to minimize the near-field crosstalk between the individual nanoantennas in the different layers (see also Supplementary Fig. 1 and Supplementary Discussion, and additional general analysis in ref. 41).

The lenses were fabricated by e-beam lithography as described in the ‘Methods' section and also in more detail elsewhere^36^. The process involves consecutive steps of lithography, plasma etching, metal evaporation and plasma-enhanced chemical vapour deposition (PECVD) of silica, which served as the dielectric spacer between the metasurfaces. Similar approaches had been used before to create three-dimensional (3D) metasurfaces for other purposes (for example, refs 41, 42). The multilayer process allows us to stack the metasurfaces with interlayer stamping precision on the order of tens of nanometres, which is crucial for the performance of some of the functions described below. Figure 1b shows the local design parameters used for the metasurface layers. The respective scanning electron microscope images of different layers are shown in Supplementary Fig. 2. One of the main advances introduced here, is the use of different metals in different layers to optimize the performance of the composite 3D metasurface. Specifically, the use of gold for the red part of the spectrum, silver for the green and aluminium for the blue is a major design advantage in that it allows us to decrease the size of the building blocks in each layer and thus to reduce the spectral crosstalk between the different layers. The disk-shaped structures were chosen because of their polarization-independent tunable plasmonic resonances. Figure 1c–e show dark-field microscope images of the individual metasurface lenses. The metasurfaces are illuminated with white light, and as can be clearly seen, each of the metasurfaces strongly scatters light at the designated colour.

We used the well-known Fresnel binary zone plate configuration^43^ for the individual lenses. The radii of the opaque concentric rings are given by:


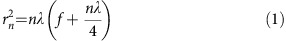


where *r*_*n*_ is the *n*th zone radius, *λ* is the wavelength and *f* is the focal distance. Rewriting equation (1) as a function of *f* gives:


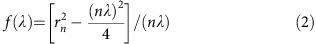


Equation (2) reveals the dispersive character of the diffractive lenses (see also Supplementary Fig. 3). For example, in the case of a conventional zone plate designed to focus green light (*λ*=550 nm) to 1 mm away from the lens, the focal plane of the entire visible spectrum spans over more than 400 μm. This chromatic aberration is the main hindrance preventing the use of such lenses for broadband or multiwavelength applications. However, by utilizing frequency selective metasurfaces, we can derive a generalized expression for *f*(*λ*) for each surface:


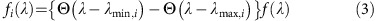


where *i* indicates the surface number, Θ is the Heaviside step function and {*λ*_min_, *λ*_max_} is the spectral band of interest. Summing over all surfaces, we can obtain the response of the multilayer composite device as follows:


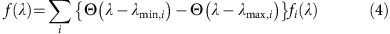


In this work, we demonstrate this approach by dividing the visible spectrum into three spectral bands.

Figure 1f shows a bright-field image of the three-layer element. Note that the zones for each colour do not fully overlap, since each layer is designed for a different colour to be focused to the same focal position of 1 mm (equation 1). In fact, the total overlap of the layers is ∼95%. This demonstrates an additional advantage of our design scheme compared to spatial multiplexing techniques^39^, where the number of subwavelength inclusions is physically limited. First, we illuminated the lens with white light (Xenon arc lamp), and measured the spectrum at the focal spot. The result is shown in Fig. 1g, where the three designed spectral RGB components at 650, 550 and 450 nm are clearly visible, thus creating the desired white focal spot, as seen in the inset of Fig. 1g. The transmission peak in the red is higher than the other two due to better fabrication and probably also larger wavelength to diameter ratio of the gold nanodiscs. The slight inaccuracies in the target wavelengths are attributed to shifts between the design dimensions and actual fabrication results.

**Figure 2:**
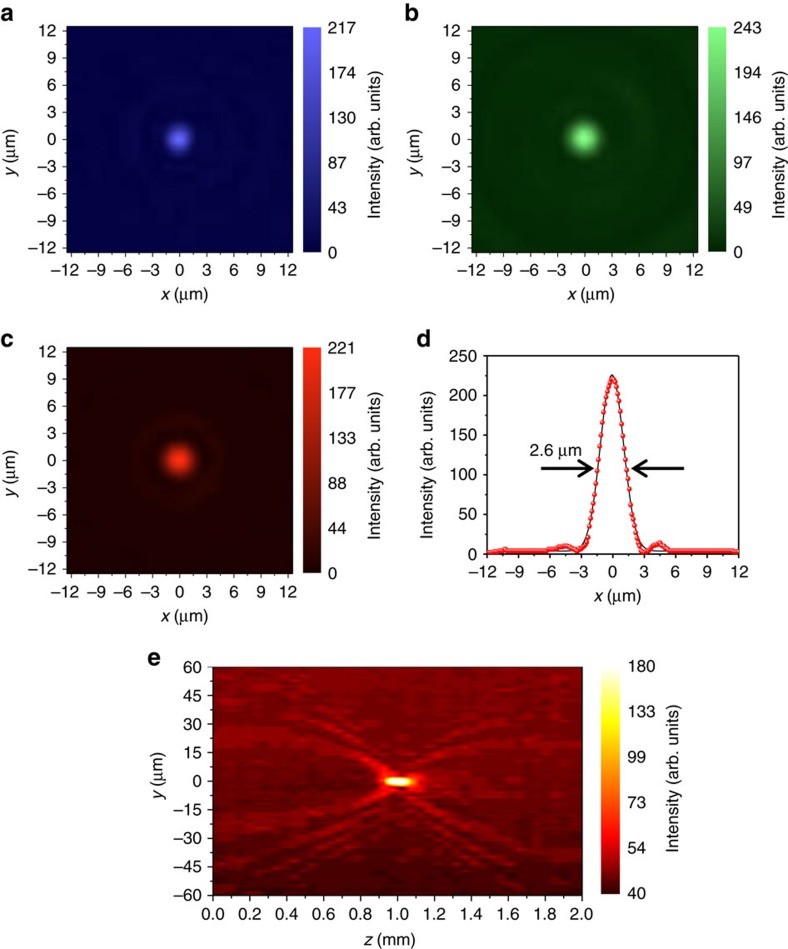
Focusing lens beam characterization with laser illumination. (**a**–**c**) Focal spots under blue (450 nm), green (550 nm) and red (650 nm) laser illuminations. (**d**) Cross section of **c**, showing the beam profile. The data points were fitted to a Gaussian profile. (**e**) Focusing of the lens under red (650 nm) laser illumination.

To characterize the focusing properties of the three individual lenses, we used laser illumination at different wavelengths (see Methods section and Supplementary Methods). The results are shown in Fig. 2a–c for illumination at 450 nm, 550 nm and 650 nm, respectively. The focal spot diameters for different colours were measured by sampling 20 points within the expected focal depth region (see Supplementary Methods and Supplementary Fig. 4). The full-width at half-maximum for each colour at the focal point is measured to be 2.6 μm, 2.43 μm and 2.11 μm for red, green and blue wavelengths, respectively (see Fig. 2d,e)—in good agreement with theoretical values. The focusing transmission efficiency was also measured for each wavelength, and found to be in the range of 5.8–8.7%, which is well within the range of the theoretical value of ∼10% for binary diffractive lenses^43^. Note that increased experimental efficiency can be obtained by optimizing the optical response of each layer, by increasing the spectral extinction ratio of each of the layers. In addition, different configurations, for example, a reflection-type phase binary zone plate, could be implemented with expected theoretical efficiencies of up to four times the efficiency of a standard binary zone plate. (see Supplementary Fig. 5 and Supplementary Discussion) and even larger efficiencies can be obtained by using impedance matching concepts^44^.

**Figure 3:**
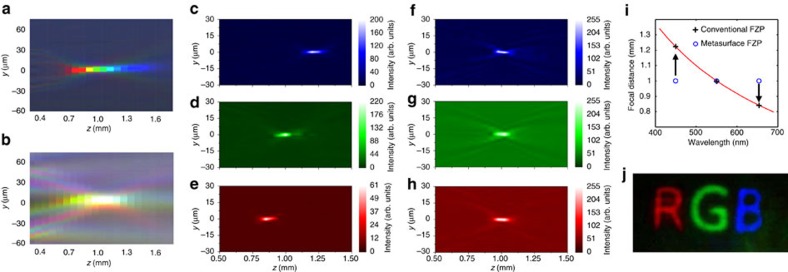
Chromatically corrected three-layer metasurface lens. Measured light focusing with conventional FZP (**a**) and metasurface FZP (**b**) under white light illumination (Xenon arc lamp, contrast normalized for viewing purposes). Chromatic aberration is apparent in **a** while the focal spot at 1 mm appears white in **b**. Images of the focal region for a conventional FZP illuminated by laser light at 450 nm (**c**), 550 nm (**d**) and 650 nm (**e**) and for the metasurface FZP (**f**–**h**), showing the aberration correction for the latter. (**i**) Theoretical calculation (equation 2) of the focal distance for a conventional FZP (red line) and the measured focal points at the RGB wavelengths of the conventional FZP (crosses) and metasurface FZP (circles). (**j**) Demonstration of colour imaging using the fabricated metasurface FZP element.

To compare the broadband operation of the new lens to a conventional binary FZP lens, we also fabricated a conventional binary FZP (see ‘Methods' section), illuminated both lenses with white light (Xenon arc lamp), and characterized the light propagation after the lenses. Figure 3a,b shows the light propagation after the conventional FZP and the multilayer metasurfaces lens, respectively. It can be seen clearly that for the case of the conventional FZP (Fig. 3a), the focus is strongly chromatically aberrated by more than 400 μm. For the multilayer metasurface lens (Fig. 3b), on the other hand, the chromatic aberrations are corrected and a white focus is formed at 1 mm away from the lens. The background of the conventional FZP is darker since it was fabricated as transparent rings in a continuous thin film, thus blocking background illumination. Also, its dynamic range is somewhat larger than the fabricated metasurfaces-based FZP that show lower extinction compared to continuous films. In Fig. 3c–e, we show the performance of conventional FZP with laser illumination (see ‘Methods' section) at wavelengths of 450 nm, 550 nm and 650 nm, respectively, and compare to the performance of the composite metasurface for the same wavelengths (Fig. 3f–h). The perfect chromatic aberration correction of the composite metasurface at these wavelengths is clear. Figure 3i depicts the measured focal distance versus wavelength for the uncorrected and corrected lenses. This measured low spread of the wavelength-dependent focal plane, and low crosstalk between the different layers, enables our lens to perform chromatic imaging, as presented in Fig. 3j (see ‘Methods' section and Supplementary Methods).

As we move away from the design wavelengths, the triplet lens shows residual chromatic aberrations due to the finite bandwidth of the plasmonic resonators (see Supplementary Fig. 3). At the design wavelengths, however, the residual power of the other wavelengths was measured to be less than 10% of the main beam. This residual crosstalk can be further decreased by minimizing fabrication errors or by designing resonators with higher-quality factors and therefore sharper linewidths. Here the advantages of our composite material approach become important. While in principle, aluminium or silver nanoresonators can be designed to cover the entire visible range and can be fabricated in a single metasurface, this task would necessitate the use of larger or more complex shapes (such as nanorods) which will be polarization sensitive. These more complex shapes may develop higher-order modes (or multiple resonances) at undesired wavelengths or larger radiative losses that would increase the residual chromatic aberration. The use of different materials for different spectral regions alleviates these problems.

### Multifunctional multilayer elements

The multilayered metasurfaces approach allows us also to multiplex several beam manipulation functionalities into a single optical element. To exploit these newly introduced capabilities, we fabricated a multilayer integrated element that can be used for super-resolution STED microscopy^45^. In STED, one laser beam with a Gaussian profile is tightly focused to excite a fluorescent sample, and a second, co-aligned doughnut-shaped beam with zero intensity at its centre depletes the emission by saturating the fluorescent transition. Thus, fluorescence is collected only from the much smaller non-depleted region. Using this method, resolution much better than the diffraction limit has been demonstrated (refs 45, 46). Typically, the optical arrangement of such a system is not trivial, involving an optical set-up for generating a doughnut-shaped beam, with a fluorescence pump beam at its centre, coupled to optics for collection of the fluorescence from the centre region, all at different wavelengths.

**Figure 4:**
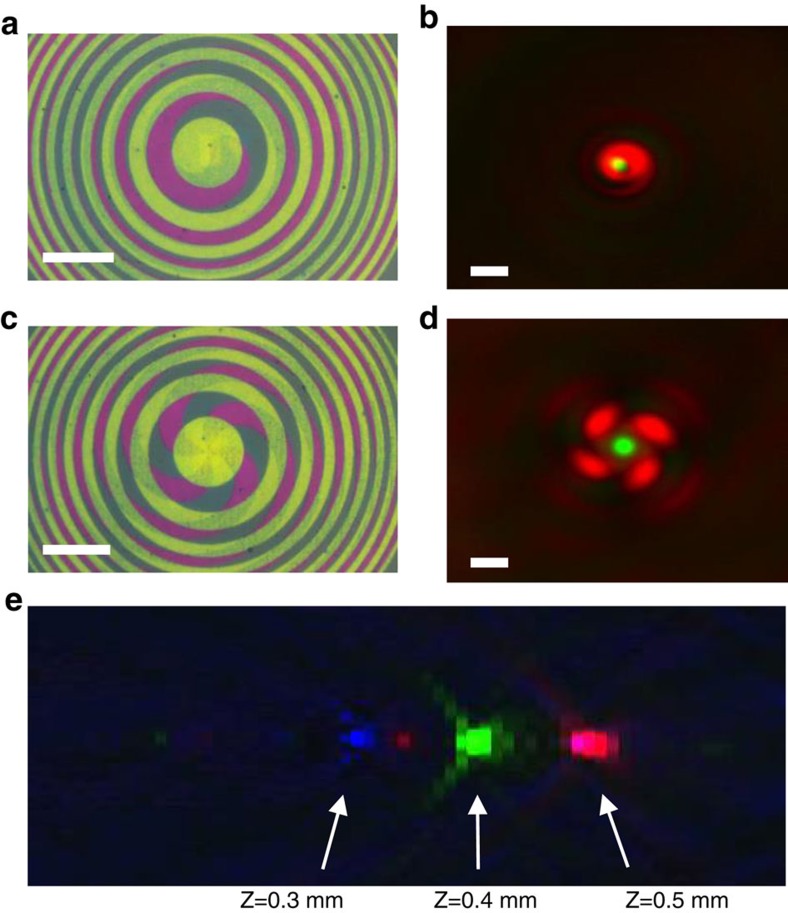
Complex lenses. (**a**) A dual-layer STED element composed of one layer designed to focus a green laser (λ=550 nm) at *f*=1 mm and a second spiral-shaped layer designed to focus a doughnut-shaped red laser beam (λ=650 nm) at the same focal distance. The topological charge of the spiral beam was set to *l*=1. (**b**) Obtained image at the focal plane for illumination with super-continuum source. (**c**,**d**) Corresponding element bright-field image and focal plane image as in **a**,**b**, respectively, for the case of topological charge *l*=4. The interference between the generated vortex beam and a background beam transmitted by the plate gives rise to the spiral-shaped vortex in **b** and the four lobe vortex in **d**. Scale bar, 35 μm (**a**,**c**) and 5 μm (**b**,**d**). See the recorded propagation of the beams in space showing their red vortex and green focusing characteristics in Supplementary Movies 1 and 2. (**e**) Demonstration of the functionality of a lens that was designed to show anomalous chromatic aberration of its RGB foci, which is shorter wavelengths focus before longer wavelengths (contrast was enhanced for clarity).

Here we use the freedom to independently choose different materials and specific designs for each layer, to demonstrate an integrated STED lens, consisting of a dual layer that tightly focuses green light with a full round beam profile, and red light to a doughnut-shaped beam at the same focal spot. We used a conventional FZP configuration for the excitation focus and fabricated on top a spiral-based FZP for the depletion beam that leads to the generation of a doughnut beam at its focus^47^ (see also Supplementary Fig. 6). Figure 4a,c shows bright-field reflection images of two such fabricated devices, where we implement lenses with different topological charges *l*=1 and *l*=4, respectively. The performances of the fabricated devices were tested with a super-continuum laser as the illumination source (see ‘Methods' section), and the results shown in Fig. 4b,d (see also Supplementary Movies 1 and 2), are in good agreement with simulated results.

The ability to design and fabricate a single optical element with different focusing features for different wavelengths opens the way to a wide range of applications. As another example, we demonstrate a lens with anomalous chromatic dispersion, where the shorter wavelengths are focused first: for example, blue, green and red colours are focused to 300 μm, 400 μm and 500 μm, respectively. The measured performance of such an anomalous lens is shown in Fig. 4e. Such an optical device could, for example, find application in the optical readout of multimedia disks, which combine Blue-Ray, DVD and CD, without the need to use beam splitters or movable lenses. This optical element thus acts as a multiband filter and a lens all-in-one device.

## Discussion

In this work, we have demonstrated a new nanotechnology-driven approach to create thin, multifunctional and spectrally multiplexed optical elements. We used this approach to demonstrate the first chromatically corrected metasurface triplet lens for RGB colours in the visible, integrated self-aligned STED elements and a diffractive lens with anomalous chromaticity. These elements are based on a multilayer design concept, where each layer is composed of nanoresonators made of the dedicated plasmonic material and designed to operate in a specific spectral band. The combined multilayer elements show complex functionalities, otherwise unachievable with conventional diffractive optics. Owing to its simple design, ease of fabrication, ultra-thin profile and low interlayer crosstalk, our multilayer, multimaterial design could find applications in future integrated opto-electronic devices, imaging systems and complex microscopy set-ups.

While the efficiencies achieved in this first ‘proof of concept' are not very high, they can, in principle, be improved by optimizing the optical response of each layer, by increasing the spectral extinction ratio of each of the layers. Furthermore, other configurations, such as reflection-type phase binary zone plates, could be considered, with expected potential efficiencies of up to four times the efficiency of a standard binary zone plate, and even larger efficiencies might be obtained by using impedance matching concepts^44^ (more details are given in the Supplementary Discussion).

Design capabilities in dual- and triple-layer configurations were demonstrated, but this concept can be extended, in principle, to any number of layers with no additional design complexity. In addition, the present paradigm can be readily implemented with other materials, building block geometries and layer designs^6^,^10^,^25^,^26^,^27^, greatly increasing the composite metasurface efficiency, covering spectral ranges beyond the visible, opening the door to hyperspectral functionality and addressing specific multifunctional optical requirements.

## Methods

### Numerical simulations

To study the resonant behaviour of the metallic nanoantennas, we used a commercially available finite difference time-domain simulation software package (Lumerical FDTD). The 3D simulation was performed with periodic boundary conditions representing a periodic array within each zone of the fabricated optical elements. To simulate the focusing properties of the lenses and the STED device, we used a beam propagation algorithm based on the transfer function in free space, which was implemented using MATLAB software. See also Supplementary Discussion.

### Sample fabrication

The samples were fabricated using multilayer e-beam lithography. We use an indium–tin–oxide covered glass as the substrate. A 200 nm thick silica layer was grown on top of the indium–tin–oxide film by PECVD. A 125 nm thick e-beam resist (PMMA 950 k A) was then spin coated and the design pattern was exposed in an electron beam lithography system (Raith eLine Plus). Alignment marks were also written to aid the stamping process of subsequent layers. After development of the resist, 30 nm of the exposed silica was etched using an inductively coupled plasma etcher (CF4 gas, 50 sccm flow, 400 W ICP power and 50 W platen power, 20 s processing time). A 30 nm thick gold film was then deposited by e-beam evaporation and subsequently lifted off in acetone in an ultrasonic bath. This first layer was covered by a 200 nm thick silica layer grown by PECVD. The process was repeated for the silver and aluminium layers. The aluminium layer was left exposed to the air, where a self-formed passivating aluminium dioxide layer prevents further degradation of the aluminium disks.

### Experimental optical set-up

We used a Zeiss (Observer Z1) inverted microscope to image the samples in transmission, reflection, bright- and dark-field modes. To measure the focusing properties, we used a home-built microscopy set-up. The emission from the sample was collected with a Mitutoyo 20X 0.42 objective, and the sample was mounted on an automated moving stage (Thorlabs Nanomax 606). We used a Xenon arc lamp as an unpolarized white light source and a femtosecond optical parametric oscillator (Chameleon OPO VIS, pulse width ∼140 fs, repetition rate 80 MHz) as the linearly polarized laser source. We used a similar set-up to study the STED element performance, with a super-continuum laser as the illumination source (NKT SuperK compact). The spectral properties of the lenses were obtained using an imaging spectrometer with a cooled back-illuminated electron multiplying charge-coupled device detector (Andor Shamrock 303i, Newton 970). The imaging of the RGB pattern was done with an LED projector and subsequent optics that were used to project the RGB pattern (Supplementary Fig. 7) to the field of view of the fabricated lens.

### Data availability

The data that support the findings of this study are available from the authors on reasonable request, see author contributions for specific data sets.

## Additional information

**How to cite this article:** Avayu, O. *et al*. Composite functional metasurfaces for multispectral achromatic optics. *Nat. Commun.* **8,** 14992 doi: 10.1038/ncomms14992 (2017).

**Publisher's note**: Springer Nature remains neutral with regard to jurisdictional claims in published maps and institutional affiliations.

## Supplementary Material

Supplementary Information Supplementary Figures, Supplementary Discussion, Supplementary Methods and Supplementary Reference

Supplementary Movie 1 Light propagation after STED element *l*=1

Supplementary Movie 2 Light propagation after STED element *l*=4
