# Supplementary material for: Composite functional metasurfaces for multispectral achromatic optics
Source: Nat Commun. 2017 Apr 5;8:14992. doi: 10.1038/ncomms14992 (PMC5382321; doi:10.1038/ncomms14992)
Supplement: Supplementary Information — Supplementary Figures, Supplementary Discussion, Supplementary Methods and Supplementary Reference [file ncomms14992-s1.pdf]

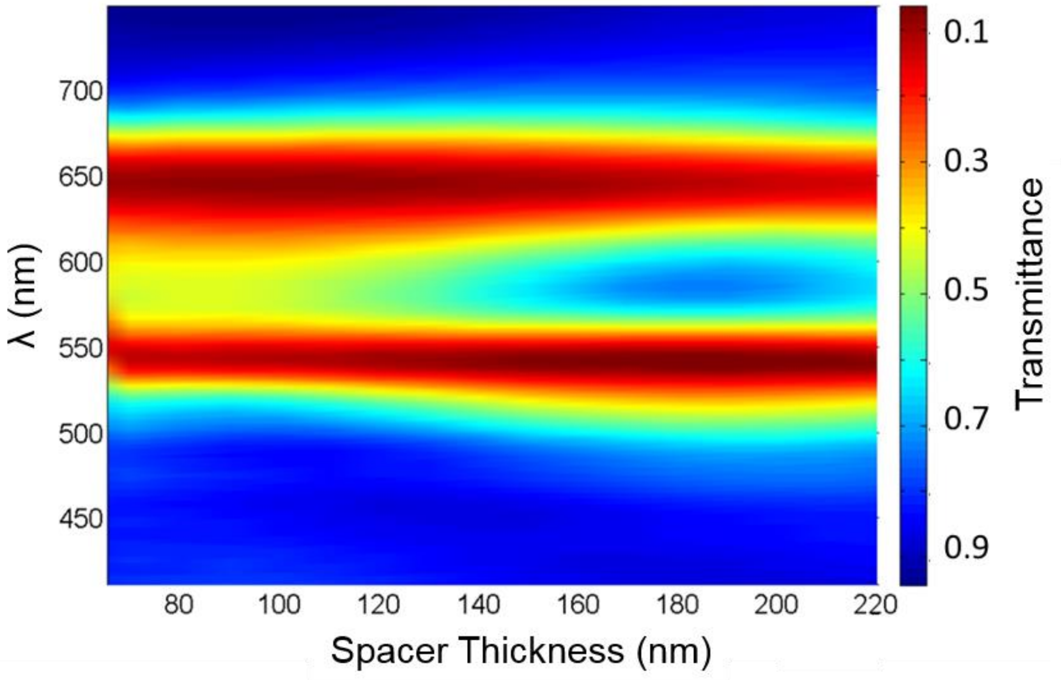


**Supplementary Figure 1. Effect of the spacer thickness on the resonance properties of the gold and silver metasurface layers**. Finite-difference time-domain calculations of the optical transmittance through the gold and silver layers spaced with $SiO_2$. At thicknesses below 90 nm, coupling effects result in changes of the resonance properties of the individual layers.

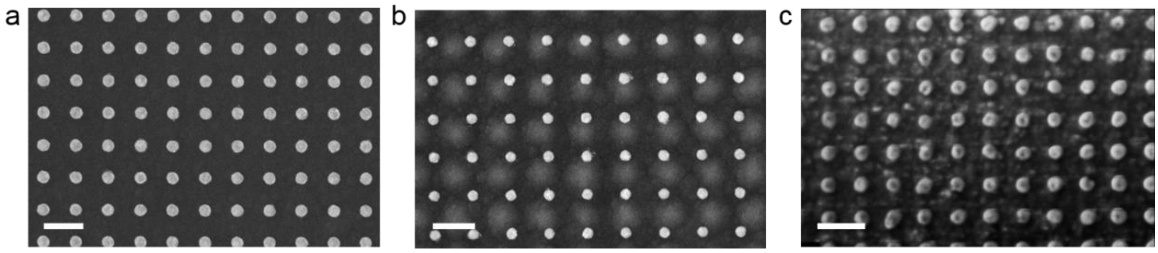


**Supplementary Figure 2. Scanning electron microscopy images (SEM) of the multilayer achromatic lens.** (a)-(c) SEM images of sections of the multi-layer lens, showing arrays of nanoparticles for the three different layers: (a) gold nanoparticles (scale bar – 400 nm), (b) silver nanoparticles (scale bar – 300 nm) and (c) aluminium nanoparticles (scale bar – 400 nm). In the silver nanoparticle layer (b), the gold nanoparticles located at the bottom layer are clearly visible in the background. The measured dimensions of each layer are ($D$ – diameter of nanoparticles, $l$ – separation between particles): $D_{Au}$=125 nm, $l_{Au}$=185 nm; $D_{Ag}$=85 nm, $l_{Ag}$=195 nm; $D_{Al}$=120 nm, $l_{Al}$=150 nm. These values slightly differ from the design values due to fabrication accuracy limitations. In addition, it can be seen that the aluminium nanoparticles (c) are less uniform in shape and size, leading to a broader, non-symmetric resonance behavior of this layer.

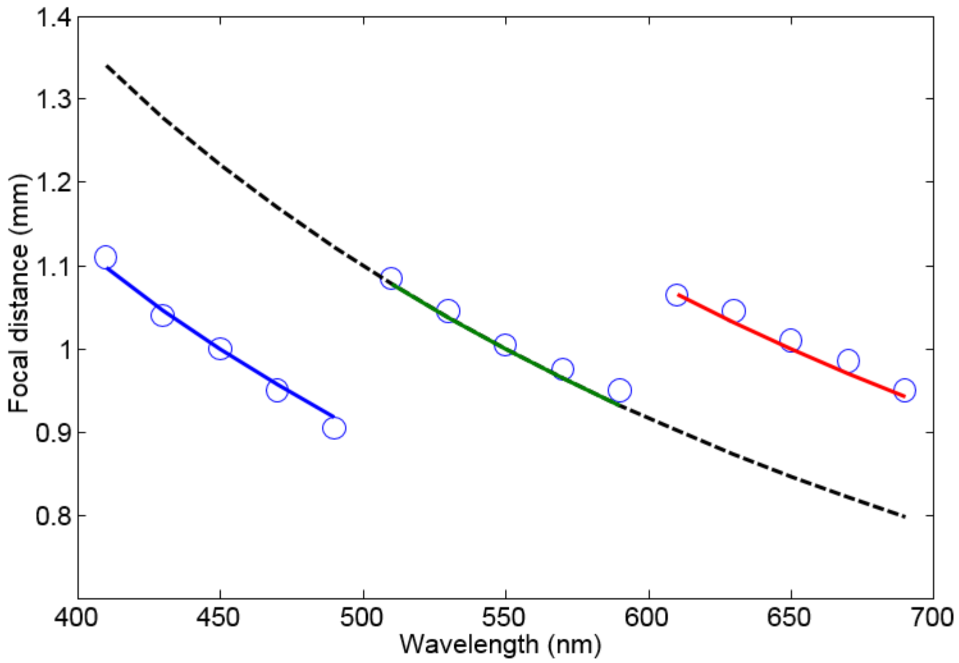


**Supplementary Figure 3**. **Chromatic dispersion of the composite metalens**. Measured focal spots across the visible spectrum (blue circles). The dashed black line shows the chromatic behaviour of a conventional Fresnel zone plate (FZP), designed to focus a green ($\lambda_G$ = 550 nm) beam at 1 mm. The blue, green and red curves represent the theoretical focal distance versus wavelength curves for three conventional FZPs, designed to focus at 1 mm wavelengths of 450 nm, 550 nm and 650 nm, respectively. Comparing the measurement to the theoretical fit of a conventional FZP (black dashed line), we see that the single curve is now divided into three curves, corresponding to $f_{1,2,3}(\lambda)$ of the three spectral bands (see Eqs. (1) – (4) in the main text for details). The plot clearly shows the significant reduction of the chromatic aberrations compared with a conventional FZP for white light illumination.

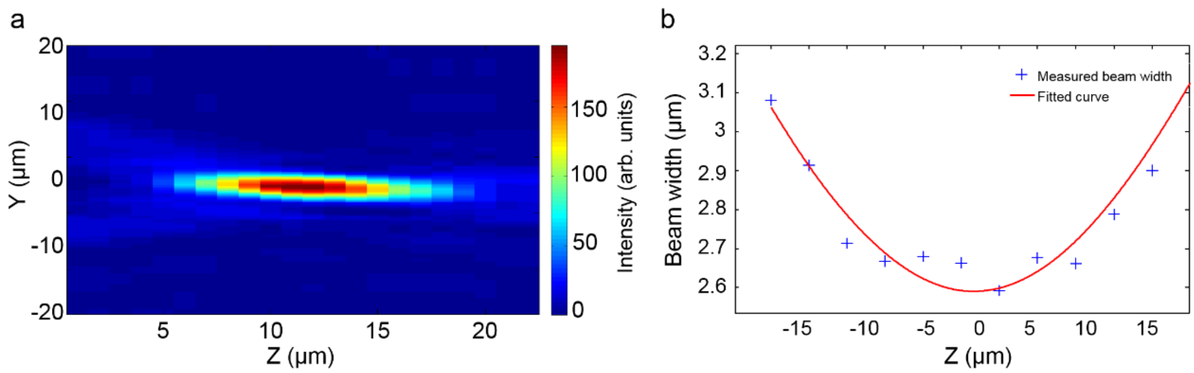


**Supplementary Figure 4. Characterization of the beam divergence of the metalens at λ = 650 nm.** (a) Focal spot divergence along the optical axis. (b) Measured Gaussian width (crosses) and the theoretical fit (red curve) to Supplementary Equation 2. The full width at half maximum of the beam (2.3 μm) was extracted at the calculated focal point.

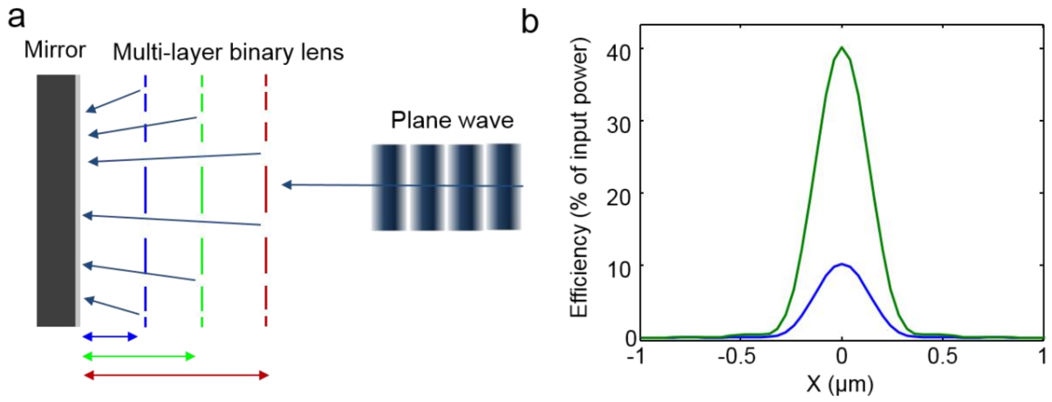


**Supplementary Figure 5. Lens design for enhanced efficiency**. (a) Illustration of an achromatic three layer phase Fresnel Zone plate (pFZP) metalens for RGB light, which uses a common reflector for the distinct layers (See Supplementary Discussion 1 for design details). (b) Cross section of the focal plane intensity, 1 mm along the *z* direction, of a conventional FZP (blue line), and a metasurface pFZP (green line), showing 4 times efficiency enhancement.

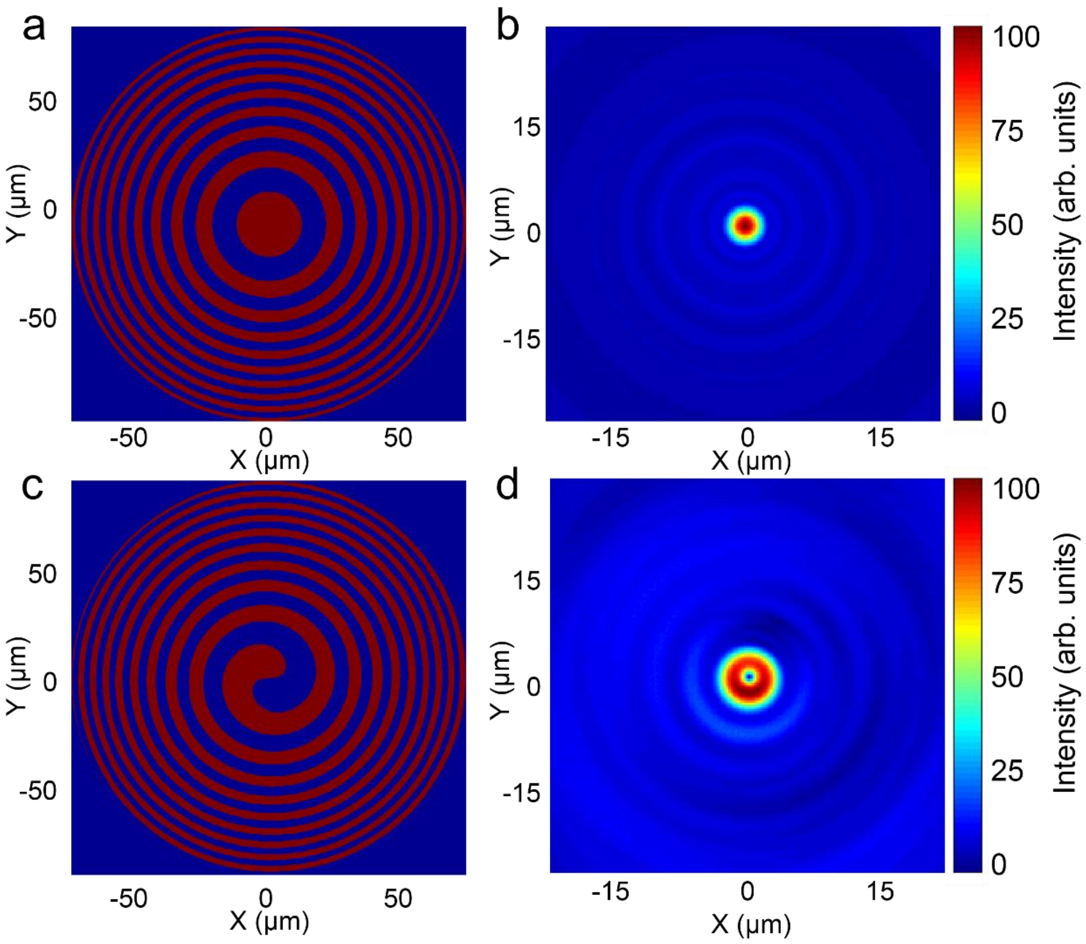


**Supplementary Figure 6. Metalenses design and simulations of the beam propagation.** Binary Fresnel zone plate (a) and the field distribution (b) at 1mm along the optical axis, for a beam at $\lambda_G$ = 550 nm. The spiral shaped mask in (c), designed for $\lambda_R$ = 650 nm, generates a doughnut shaped beam at the focal spot, as seen in (d) (See Supplementary Discussion 2).

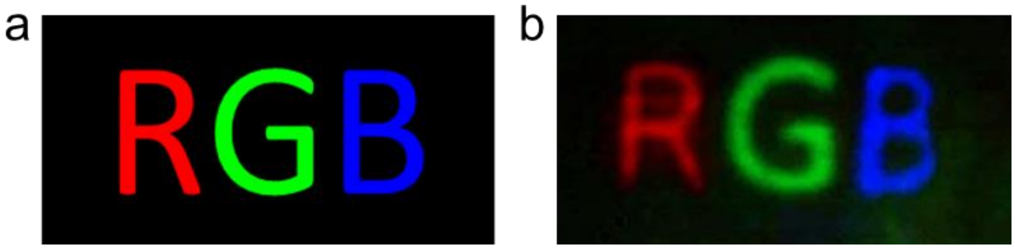


**Supplementary Figure 7. Imaging with the metasurface lens**. (a) RGB projected pattern, which serves as the object to be imaged by the composite metalens. (b) Image collected by the colour CCD. The projected image was first resized using a 4*f* system to fit the numerical aperture of the metasurface lens (See Supplementary Method 3). The image formed by the lens was then magnified by an objective (20X) and imaged on the CCD.

## Supplementary Discussion

*Dielectric spacer thickness calculations*

In order to minimize crosstalk between layers, we used a 200 nm $SiO_2$ spacer between layers. Supplementary Figure 1 shows a finite-difference time-domain simulation result of the optical transmission through two such layers, in this case Au and Ag, spaced with $SiO_2$. It can be seen that as we decrease the spacer thickness below 90 nm, coupling effects start to be more significant. Between 70 nm and 90 nm we do not see a large shift in the resonance of either nanoparticle, but the width of the peaks, and extinction amplitude changes are clearly visible. Similar numerical tests performed on the Al layer and the Ag layer, have shown very similar behaviour, with no cross coupling above 90 nm spacing. Therefore, we estimate that any spacer thickness down to 100 nm should perform similarly. Larger thicknesses could result in unwanted cavity effects. However, in our case, as the spectral crosstalk between the different layers is less than 10%, these cavity effects will not modify considerably the overall response. We note that in the experiment, the selected thickness of 200 nm performed well, and did not experimentally test other spacer thicknesses.

*Lens design for enhanced efficiency*

In order to enhance the efficiency and imaging properties of binary lenses, different designs have been suggested, including phase zone plates, azimuthally modified zone plates (e.g. the Beynon Gabor zone plate), and photon sieves[1]. It is well known for example, that changing the opaque zones in a conventional zone plate to transparent zones that provide an additional π phase shift will result in increased efficiency at the primary foci to ~40%. This device is called phase Fresnel zone plate (pFZP). Supplementary Figure 5a illustrates a possible implementation of a 3 layer reflection type pFZP based on our multi-layer metasurface design scheme. As in our binary FZP design, each layer focuses a different part of the spectrum. In this design however, the distance of each layer from the mirror (coloured double-sided arrows), is calculated to impose a roundtrip π phase shift relative to the backward scattered plane wave, effectively creating a pFZP. Supplementary Figure 5b shows the simulated cross section of the optical field intensity at the primary focal spot (simulated by beam propagation method in MATLAB), comparing the efficiency at the primary focus of a conventional binary FZP and the proposed metasurface pFZP, clearly showing 4 times increase in efficiency.

*Metalenses design and beam propagation simulations*

To analyse the wavelength-scale performance of our devices, we simulated the optical waves using a Fourier beam propagator, implemented in MATLAB. In the simulation, we assumed a Gaussian beam normally incident on the device. The simulated device and the optical field at the focal point are presented in Supplementary Figure 6.

To generate a doughnut shaped beam for the STED (stimulated emission depletion) element, we used a spiral-shaped binary structure according to the following relation

$$mask = \frac{1}{2}\{1 + sign\left(\cos(\left(k\frac{r^2}{2f}\right) + l\theta\right))\} \quad (1)$$

where $f$ is the focal distance, $\theta$ and $r$ are the polar coordinates of the system, and $l$ is the topological charge. We generated two spiral masks, with topological charges of $l = 1$ (Supplementary Figure 6c) and $l = 4$.

This structure resembles a conventional zone plate that focuses light to the designed focal point, with the exception of a phase singularity along the optical axis which comes from the spiral structure. This creates the desired doughnut beam at the focal point as shown in Supplementary Figure 6d.

The STED element is therefore composed of two layers: The first layer is composed of Au nanodiscs, designed to interact with red light, around 650 nm, and focus it into a doughnut beam at 1 mm along the optical axis. The second layer is composed of Ag nanodiscs, designed to interact with green light, around 550 nm, and focus it to 1 mm along the optical axis. All dimensions, i.e. spacer thickness, nanodiscs diameters and inter-particle spacing are identical to the ones used for the 3 layer metasurface lens (see fig. 1 in the main text for dimension details).

## Supplementary Methods

### *Focal spot characterisation*

To determine the focal plane and the beam diameter at this plane, we sampled 20 points along the propagation direction near the expected focal region, and measured the width of the Gaussian beam at each plane. We fitted the measured points to the theoretical propagation of a Gaussian beam along the optical axis, which is given by:

$$\omega(z) = \omega_0\sqrt{1 + (\frac{z}{z_R})^2} \tag{2}$$

where $\omega_0$ is the beam waist, and $z_R$ is Rayleigh range. The measured focused beam and the fit to the data points for the red beam at 650 nm are plotted in Supplementary Figure 4.

### *Lens efficiency calculation*

We define the efficiency of the binary Fresnel zone plate as the total power at the primary focal spot relative to the total power incident upon the lens. We used a calibrated CMOS camera to measure both. We used the method described in Supplementary Method 1 to find the focused beam waist. The focused beam was fitted to a Gaussian profile and the power was then measured at the beam waist, covering approximately 86% of the total power.

### *Imaging with the metasurface lens*

We used a commercial projector and a 4*f* system to project a digital image of size comparable to the lens size (~500 μm), to the field of view of the fabricated multi-layer lens. The image formed by the lens was then collected with an objective lens (Mitutoyo 20X 0.42), and then imaged on a colour CCD. The original projected image and the image formed on the CCD are given in Supplementary Figure 7.

## Supplementary Reference

1. Vierke, T. & Jahns, J. Diffraction theory for azimuthally structured Fresnel zone plate. *J. Opt. Soc. Am. A. Opt. Image Sci. Vis.* **31,** 363–372 (2014).
